# Health-related quality of life after open and robot-assisted radical prostatectomy in low- and intermediate-risk prostate cancer patients: a propensity score-matched analysis

**DOI:** 10.1007/s00345-020-03144-9

**Published:** 2020-03-04

**Authors:** Alexander Kretschmer, Robert Bischoff, Michael Chaloupka, Friedrich Jokisch, Thilo Westhofen, Philipp Weinhold, Frank Strittmatter, Armin Becker, Alexander Buchner, Christian G. Stief

**Affiliations:** Department of Urology, Ludwig-Maximilians-University, Marchioninistrasse 15, 81377 Munich, Germany

**Keywords:** EORTC QLQ-C30, Open radical prostatectomy, Robot-assisted radical prostatectomy, Health-related quality of life

## Abstract

**Purpose:**

Introduction of robot-assisted radical prostatectomy (RARP) has revolutionized the therapeutic landscape of organ-confined prostate cancer (PCa). However, comparative analyses focused on health-related quality of life (HRQOL) after RARP and open retropubic prostatectomy (ORP) are sparse.

**Methods:**

In the current retrospective analysis,inclusion criteria encompassed PSA ≤ 10 ng/ml, ≤ pT2c, ISUP ≤ 3, age ≤ 65 years, and preoperative continence. A propensity score-matched patient cohort [*n* = 418 (ORP: 209, RARP: 209)] was created and HRQOL was prospectively assessed based on validated questionnaires (EORTC QLQ-C30) preoperatively, 3 months, 12 months, and 24 months postoperatively. Primary endpoint was good general HRQOL based on previously published cut-off values. Erectile function was measured via IIEF-5, urinary continence via ICIQ-SF questionnaire. Multivariable analysis included binary logistic regression models (*p* < 0.05).

**Results:**

Open retropubic prostatectomy and RARP cohorts were well balanced. General HRQOL was significantly higher for ORP compared to RARP after 3 months (70.1 vs. 61.6, *p* = 0.001), but not at the remaining follow-up time points. There were no significant differences for the remaining QLQ-C30 functioning and symptom scores. In multivariable analysis stratified for IIEF-5 and ICIQ-SF scores and surgeon experience, RARP could be confirmed as a marginally independent predictor for lower ratios of good general HRQOL after 3 months (OR 0.464, 95% CI 0.215–0.999; *p* = 0.050) without any differences at the remaining time points.

**Conclusions:**

The current study addresses various HRQOL outcomes over a postoperative period of up to 2 years in a homogenous propensity score-matched contemporary cohort. Marginally better general HRQOL outcomes could be detected for ORP compared to RARP 3 months postoperatively.

**Electronic supplementary material:**

The online version of this article (10.1007/s00345-020-03144-9) contains supplementary material, which is available to authorized users.

## Introduction

Introduction of robot-assisted radical prostatectomy (RARP) has already revolutionized the therapeutic landscape of organ-confined prostate cancer over the last decade. However, the absence of level 1 evidence regarding superiority of the robot-assisted approach has led to distinct controversies [1]. The highest grade comparative evidence to date is provided by the well-anticipated randomised trial by Coughlin et al. who analysed patient-reported outcomes (PROMs) of a total number of 296 patients after a follow-up of 24 months [2]. The authors did not observe significantly different outcomes between open retropubic prostatectomy (ORP) and RARP based on validated questionnaires assessing postoperative urinary and sexual function as well as health-related quality of life (HRQOL). Regarding oncological outcomes, the authors found a biochemical recurrence rate of 9% for ORP compared to 3% for RARP, but stressed out that postoperative oncological management was not standardized between the participating centres [2]. Similarly, a recent meta-analysis concluded that there is no evidence regarding the comparative effectiveness of RARP compared with ORP for oncological outcomes can be drawn yet [3]. In a recent comparative study including more than 10,000 radical prostatectomies (RP), no statistically significant differences regarding 48-month BCR could be observed [4]. In the absence of profound oncological data, a distinct focus on PROMs seems crucial. In the current study, we provide short- and mid-term HRQOL and functional data from a large propensity score-matched patient cohort with low- and intermediate-risk localized prostate cancer that underwent ORP or RARP by experienced high-volume surgeons.

## Patients and methods

### Patient population, study design and data assessment

To be eligible for the current study, patients had to fulfil the following inclusion criteria: PSA ≤ 10 ng/ml, ≤ pT2c, ISUP ≤ 3, age ≤ 65 years, no clinical lymph node involvement, no clinical indication for metastatic disease based on preoperative bone scan or CT scan, and surgery performed by experienced surgeons with a minimum of 50 previous cases. In addition, preoperative continence was mandatory to warrant inclusion.

Between March 2014 and March 2018, 2419 radical prostatectomies (*n* = 898 RARP, *n* = 1521 ORP) have been performed in 1 tertiary care centre.

After approval by an institutional review board, PROMs were prospectively retrieved preoperatively, 3 months postoperatively, 12 months postoperatively, or 24 months postoperatively. Hereby, questionnaires were sent per mail to eligible patients. Erectile dysfunction was assessed via the validated International Index of Erectile Function (IIEF-5) questionnaire. Good erectile function was defined as IIEF-5 score of ≥ 18.

Patients that met all inclusion criteria were retrospectively selected and consequently a propensity score matching including the variables “age at prostatectomy” as well as “PSA level (ng/ml) at prostatectomy”, and. “pT stage” was performed. Hereby, a matched cohort of 418 patients (*n* = 209 RARP, *n* = 209 ORP) was created and further analysed.

Urinary continence was assessed using the International Consultation on Incontinence Questionnaire in its short form (ICIQ-SF). The ICIQ-SF is a three-item validated questionnaire. The total score ranges from 0 to 21, with higher scores indicating greater severity of urinary incontinence [5].

HRQOL was assessed using the EORTC QLQ-C30 questionnaire. The primary endpoint general HRQOL was assessed based on the global health status (GHS) domain of the QLQ-C30 questionnaire (questions 29 and 30) following current EORTC instructions [6]. Following Snyder et al. [7] good general HRQOL was defined as GHS of ≥ 70. For GHS, higher scores represent better general HRQOL. For the QLQ-C30 functioning scores, higher scores represent better functioning. For QLQ-C30 symptom scores, higher scores represent greater impact of the respective symptom.

### Surgical procedure

The nerve-sparing procedure for ORP at our institution has been described before [8, 9] and did not vary substantially from the procedure that was used for RARP. Nerve-sparing procedure was performed when optimal surgical outcome was not compromised. Posterior urethral reconstruction was performed as described by Rocco et al. [10, 11] for both ORP as well as RARP. Surgical techniques of the vesicourethral anastomosis varied slightly between the open and robotic approach. For ORP, five separate outside–in single-knot sutures were applied. For RARP, the principles described by van Velthoven et al. [12] were followed.

### Statistical analysis

As indicated above, a propensity score matching was performed and a matched patient cohort was created. Comparisons of PROMs as well as EORTC QLQ-C30 subdomains between the RARP and ORP subgroup were performed using Kruskal–Wallis analysis of variance and post hoc testing whenever denoted. For categorical data, Fisher’s exact test and Chi-square test were used. Primary endpoint for univariate and multivariable analyses was good general HRQOL at the respective time based on a GHS score of ≥ 70, following previously published cut-off values [7]. For multivariable analysis, a binary logistic regression model was used. Here, the number of events was defined as the number of patients with a GHS score of 70 or more. All statistical analyses were performed using SPSS V25.0 (IBM, Armonk, NY, USA). A *p* value of < 0.05 was considered to be statistically significant.

## Results

### Perioperative patient characteristics, functional and oncological outcomes

Patient characteristics are summarized in Table 1. Briefly, ORP and RARP cohorts were well balanced regarding patient-derived factors age, body mass index, and prostate volume as well as functional (IIEF-5, ICIQ-SF) and oncological baseline values.

**Table 1:** Patients characteristics of 418 patients after open retropubic (ORP) and robot-assisted radical prostatectomy (RARP) included in the current study

	ORP	RARP	*p*
No. of patients	209	209	
Age [years; mean ± SD]^a^	58.3 ± 4.8	58.8 ± 4.6	0.929
BMI [kg/m^2^; mean ± SD]	26.9 ± 3.4	27.1 ± 3.7	0.732
PSA preop. [ng/ml; mean ± SD]^a^	6.2 ± 2.2	5.9 ± 2.4	0.320
IIEF-5 preop. 18 or more [*n* (%)]	149 (71.3)	158 (75.6)	0.262
ICIQ-SF preop. [mean ± SD]	0.8 ± 2.6	0.7 ± 2.1	0.978
IPSS preop. [mean ± SD]	7.2 ± 6.2	6.9 ± 5.5	0.985
Prostate volume [ml; mean ± SD]	49.5 ± 16.8	49.6 ± 15.0	0.603
Nerve-sparing procedure [*n* (%)]	199 (95.2)	197 (94.3)	0.760
pT stage [*n* (%)]			
pT2a	18 (8.6)	26 (12.4)	0.146
pT2b	9 (4.3)	4 (1.9)	
pT2c	182 (87.1)	179 (85.6)	
ISUP [*n* (%)]			
1	53 (25.4)	52 (24.9)	0.648
2	110 (52.6)	118 (56.5)	
3	46 (22.0)	39 (18.7)	
PSM [*n* (%)]	30 (14.4)	21 (10.1)	0.231

*BMI* body-mass index, *ICIQ-SF* International Consultation of Incontinence Short Form, *IIEF-5 *International Index of Erectile Function, *IPSS *International Prostate Symptoms Severity Score, *ISUP *International Society of Urological Pathology, *PSA *prostate specific antigen, *PSM *positive surgical margin, *SD *standard deviation

^a^Propensity score-matched variables

Patients were only classified as “nerve-sparing” if bilateral nerve-sparing has been performed. In total, only five patients underwent unilateral nerve-sparing. Thus, no separate analysis of this patient subcohort has been performed.

Response rates from eligible patients were 62.2% after 3 months, 60.7% after 12 months, and 55.7% after 24 months. At least one follow-up at any respective time point was available for every patient included in the current study. Response rates did not vary significantly after stratification for surgical approach [3 months: 62.7% (ORP) vs. 61.7% (RARP; *p* = 0.920); 12 months: 55.9% vs. 65.3% (*p* = 0.163); 24 months: 51.9% vs. 59.4% (*p* = 0.122)].

In total, six surgeons (3 × ORP, 3 × RARP) met the surgical experience inclusion criteria of the current analysis. Median pre-analysis caseload for ORP surgeons was 115 (range 57–2248), median pre-analysis caseload for RARP surgeons was 72 (range 60–502).

Functional outcomes are summarized in supplementary Table 1. Briefly, good erectile function rates were slightly in favour of the RARP compared to the ORP subgroup. After 24 months, 64.3 (ORP) and 73.8% (RARP) of the patients with a preoperative IIEF-5 score of 18 or more had regained good sexual function (*p* = 0.190). After 3 months, we found slightly better continence results for ORP compared to RARP based on the respective mean ICIQ-SF total scores (3.9 vs. 5.3; *p* = 0.003). 12 months and 24 months postoperatively, ICIQ-SF scores declined for both groups with no significant differences regarding urinary continence between ORP and RARP.

As indicated in Table 1, positive surgical margin rates were slightly higher in the ORP subgroup (14.4 vs. 10.1%, *p* = 0.231). Within the follow-up period, radiotherapy was performed in 12 patients (ORP: 6; RARP: 6), 5 patients were under ongoing androgen deprivation therapy (ORP: 2; RARP: 3). There was no cancer-related death.

### Health-related quality of life

Analysis of the functioning and symptoms subdomains as well as financial difficulties and general HRQOL based on global health status is summarized in Table 2.

**Table 2:** Mean EORTC QLQ-C30 scores as well as standard deviations (SD) after open retropubic (ORP) and robot-assisted radical prostatectomy (RARP)

	Mean (SD) EORTC QLQ-C30 score											
	Preoperative			3-month follow-up			12-month follow-up			24-month follow-up		
	ORP	RARP	*p*	ORP	RARP	*p*	ORP	RARP	*p*	ORP	RARP	*p*
Symptom scale												
Dyspnoea	3.6 (12.6)	6.3 (17.8)	0.070	9.9. (15.4)	9.2 (18.9)	0.295	10.3 (23.7)	3.5 (10.5)	0.329	6.4 (18.9)	10.1 (18.6)	0.246
Pain	8.1 (18.0)	9.4 (18.6)	0.363	12.8. (16.8)	17.2 (23.7)	0.352	9.2 (17.0)	7.9 (17.0)	0.687	12.3 (18.9)	9.4 (18.7)	0.329
Fatigue	11.6 (17.1)	14.5 (20.3)	0.452	20.7 (20.6)	25.4 (25.4)	**0.013**	13.4 (18.4)	19.9 (24.7)	0.400	16.2 (20.4)	18.8 (22.8)	0.736
Insomnia	16.9 (26.4)	24.2 (32.2)	**0.044**	21.6 (29.6)	27.3 (30.6)	0.367	13.8 (24.4)	19.8 (29.9)	0.220	26.8 (31.2)	24.6 (33.7)	0.354
Appetite loss	2.2 (9.6)	3.4 (13.3)	0.472	2.7 (12.1)	3.0 (9.7)	0.580	0.9 (8.5)	1.2 (9.6)	1.000	1.3 (6.5)	2.9 (9.6)	0.484
Nausea/vomiting	0.6 (3.2)	1.0 (6.6)	0.826	0.9 (5.5)	3.1 (12.1)	0.260	3.4. (9.3)	0.9 (3.8)	0.330	4.5 (13.0)	3.6 (11.2)	0.930
Constipation	3.6 (13.9)	3.9 (14.6)	0.821	12.6 (25.3)	9.1 (17.2)	0.844	6.9 (13.7)	7.0 (17.8)	0.744	7.7 (19.6)	5.8 (12.9)	0.950
Diarrhoea	5.1 (14.3)	7.8 (18.3)	0.123	7.2 (13.9)	10.1 (21.2)	0.747	4.6 (11.7)	7.1 (17.7)	0.796	11.5 (18.7)	7.2 (17.3)	0.310
Financial difficulty scale	2.6 (10.7)	4.5 (16.0)	0.271	13.5 (25.4)	16.2 (25.4)	0.545	7.8 (12.9)	7.0 (17.8)	0.829	6.7 (13.5)	9.5 (16.3)	0.185
Function scale												
Physical	96.8. (8.6)	95.8 (9.5)	0.296	92.1 (9.4)	86.5 (16.6)	0.334	93.3 (13.2)	95.1 (10.1)	0.753	93.8 (10.8)	93.0 (11.3)	0.645
Role	94.0 (17.2)	91.8 (18.5)	0.094	77.9 (21.9)	66.1 (18.2)	**0.002**	87.4 (17.1)	80.7 (23.7)	0.376	86.5 (22.6)	81.9 (20.7)	0.217
Cognitive	91.7 (15.0)	90.7 (15.8)	0.583	86.0 (17.8)	83.3 (23.9)	0.954	92.0 (14.5)	90.4 (17.0)	0.762	88.5 (18.1)	87.0 (16.6)	0.583
Emotional	72.4 (23.6)	71.0 (23.3)	0.493	73.6 (22.9)	64.9 (21.8)	0.083	81.3 (20.0)	71.5 (27.7)	0.289	75.0 (25.4)	76.8 (24.4)	0.823
Social	87.2 (20.4)	86.6 (19.4)	0.457	71.2 (27.1)	64.6 (29.1)	0.325	80.5 (23.2)	78.1 (27.2)	0.813	71.0 (25.0)	75.4 (25.3)	0.470
Global health status	74.7 (19.7)	71.6 (20.2)	0.115	70.1 (19.3)	61.6 (19.2)	**0.001**	75.7 (19.1)	70.1 (19.8)	0.069	71.6 (21.6)	72.2 (21.9)	0.831

Bold and underline values indicate *p* < 0.05

**Fig. 1:**
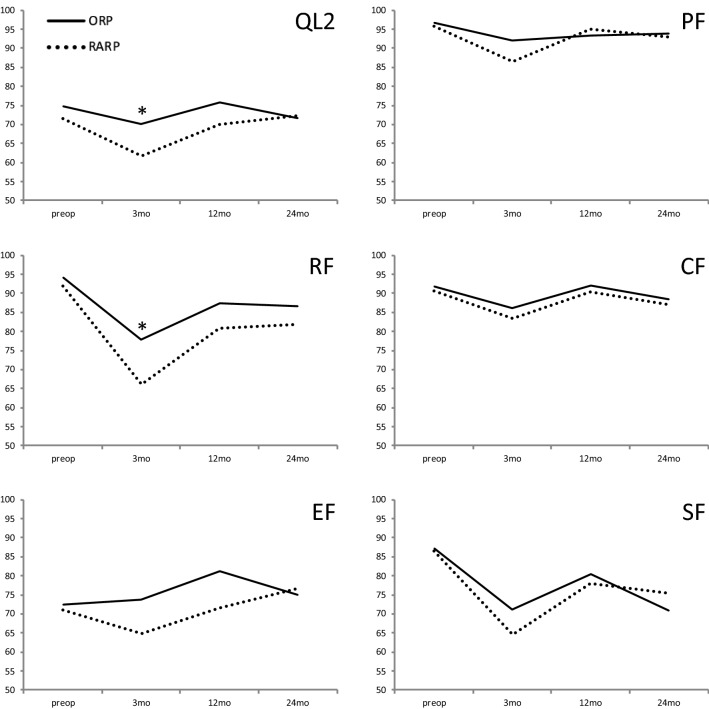
Global health status (QL2) as well as functioning scores based on the EORTC QLQ-C30 questionnaire preoperatively, 3 months (mo), 12 months, and 24 months after open retropubic prostatectomy (ORP) or robot-assisted radical (RARP) prostatectomy. *p < 0.05. *PF *physical functioning, *RF* role functioning, *CF* cognitive functioning, *EF* emotional functioning, *SF* social functioning

Regarding general HRQOL, we found significantly higher mean values for ORP compared to RARP after 3 months (70.1 vs. 61.6, *p* = 0.001), but not after the remaining follow-up periods. Consequently, 55.7 (OPR) vs. 29.5% (RARP) had a good general HRQOL based on pre-specified cut-off values after 3 months (*p* < 0.001), with no significant differences regarding the respective ratios at the remaining time points [7] (supp. Table 1). Regarding the longitudinal development of postoperative general HRQOL, we found a decline during the short-term postoperative period that increased and reached baseline values during the longer-term follow. Similar curves were found for the functioning subdomains of the QLQ-C30 questionnaire (Fig. 1). Hereby, no relevant differences except for 3-month role functioning [77.9 (ORP) vs. 66.1 (RARP); *p* = 0.002] were found between the ORP and RARP patient subcohort.

**Fig. 2:**
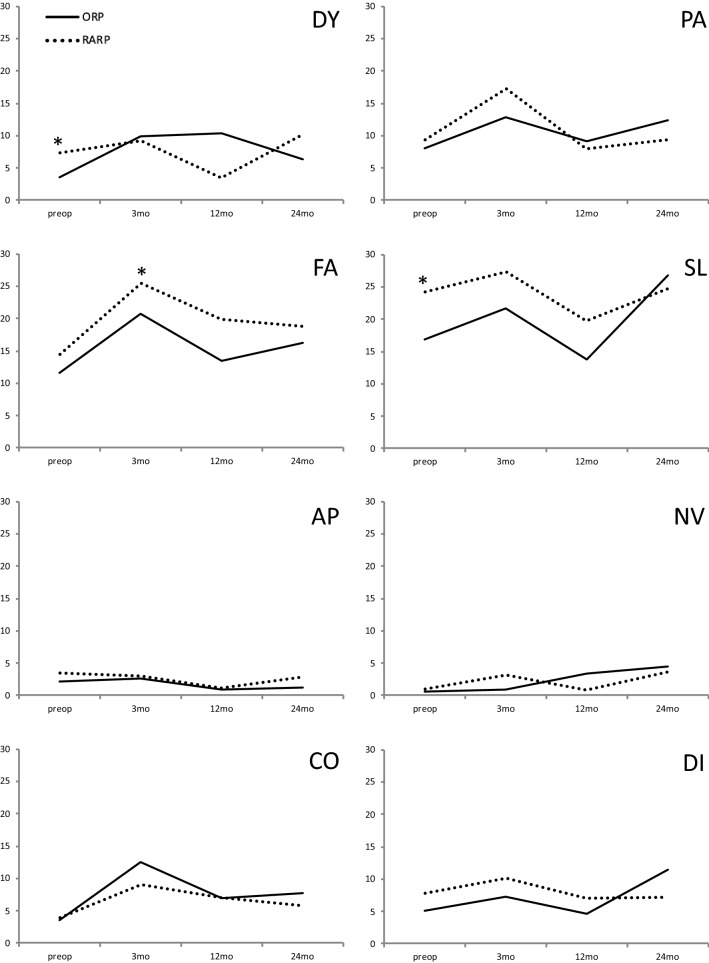
Symptom scores based on the EORTC QLQ-C30 questionnaire preoperatively, 3 months (mo), 12 months, and 24 months after open retropubic prostatectomy (ORP) or robot-assisted radical (RARP) prostatectomy. *p < 0.05. *DY *dyspnea, *PA *pain, *FA *fatigue, *SL* insomnia, *AP* appetite loss, *NV* nausea/vomiting, *CO* constipation, *DI* diarrhea

Regarding symptom scores, we found significantly increased preoperative levels of insomnia for RARP compared to ORP patients (24.2 vs. 16.9; *p* = 0.044) as well as significantly higher fatigue levels for RARP compared to ORP patients (25.4 vs. 20.7; *p* = 0.013). No further significant differences between the two subgroups were observed (Fig. 2).

Since HRQOL is vastly affected by continence as well as erectile function outcomes, a multivariable analysis stratified for respective IIEF-5 as well as ICIQ-SF scores was performed. In addition, to account for different experience levels of the participating surgeons, a sensitivity analysis focusing on the most experienced ORP and the most experienced RARP surgeon compared to all remaining surgeons was included (Table 3). Hereby, RARP could be confirmed as an independent predictor for lower ratios of good general HRQOL (defined by previously published cut-off values [7]) after 3 months with a marginally significant *p* value of 0.050. Due to small number of events, the influence of adjuvant therapies was not separately analysed.

**Table 3:** Multivariable analysis for predictive features of good health-related quality of life after open retropubic and robot-assisted radical prostatectomy (RARP)

Follow-up	Predictive feature	Regression coefficient	Odds ratio	95% CI	*p* value
3 months	ICIQ-SF (score)	− 0.264	0.768	0.691–0.853	**< 0.001**
	Surgeon experience (all vs. most experienced)	− 0.179	0.836	0.385–1.815	0.651
	IIEF-5 (score 18 or more)	0.011	1.011	0.324–3.149	0.985
	RARP	− 0.768	0.464	0.215–0.999	**0.050**
12 months	ICIQ-SF (score)	− 0.272	0.762	0.660–0.880	**< 0.001**
	Surgeon experience (all vs. most experienced)	− 0.410	0.664	0.276–1.597	0.360
	IIEF-5 (score 18 or more)	2.130	8.417	2.280–31.078	**0.001**
	RARP	− 0.549	0.578	0.240–1.389	0.220
24 months	ICIQ-SF (score)	− 0.420	0.657	0.499–0.865	**0.003**
	Surgeon experience (all vs. most experienced)	− 1.221	0.295	0.067–1.289	0.105
	IIEF-5 (score 18 or more)	2.101	8.176	1.683–39.729	**0.009**
	RARP	0.548	1.729	0.435–6.877	0.437

Bold and underline values indicate *p* < 0.05

Endpoint (event) was good general health-related quality of based on a QLQ-30 global health status score of 70 or more

*CI* confidence interval, *ICIQ-SF *International Consultation of Incontinence Short Form, *IIEF-5 *International Index of Erectile Function

## Discussion

Despite widespread use of RARP in the surgical therapy of localized as well as locally advanced prostate cancer, level 1 evidence supporting its use is still lacking. Consequently, current EAU guidelines state that both open as well as laparoscopic (conventional or robot-assisted) approaches are equivalent as long as they are performed by skilled surgeons [13]. In the absence of profound oncological data, soft endpoints such as HRQOL are essential to assess potentially beneficial effects of competing surgical techniques.

The prospective controlled LAPPRO (laparoscopic prostatectomy robot open) trial assessed short-term PROMs from more than 2500 patients and focused on perioperative characteristics, complications, and hospital readmission rates [14] as well as urinary and sexual function outcomes [15]. After 12 months of follow-up, the authors found similar incontinence rates of 21.3% (RARP) and 20.2% (ORP) between the surgical approaches. However, it has to be emphasized that definitions of continence as well as methods of continence function data retrieval vary between the currently available studies. In the current study, we provide continence data based on the validated and objective ICIQ-SF questionnaire. Regarding sexual dysfunction, similar results between the current study and the LAPPRO trial were reported [15].

In a recent study including almost 1500 patients with follow-up available, community-based outcomes after ORP and RARP based on the CaPSURE database were assessed and a self-reporting HRQOL questionnaire was included. The authors did not find significant differences regarding HRQOL outcomes after completion of a follow-up of up to 3 years [16]. Notably, in line with our findings, urinary continence rates within the first year of follow-up were superior in the ORP compared to the RARP subgroup. However, there were significant differences regarding tumour stage, Cancer of the Prostate Risk Assessment score, and Gleason grade between the ORP and the RARP subgroup [16]. In contrast, our cohort consists of two propensity score-matched, well-balanced subgroups without significant differences regarding tumour stage, PSA level, and age at radical prostatectomy.

In a well-anticipated randomized trial, Coughlin et al. [2] randomized 326 patients of whom 163 underwent RARP and found similar functional as well as oncological outcomes. However, it has to be stated that the follow-up period was too short to draw any meaningful conclusions regarding the oncological outcomes. Furthermore, the surgeon who performed the open retropubic prostatectomies was more experienced compared to the surgeons who performed RARP. Thus, it has been debated that one of the main findings of the trial was a relatively steeper learning curve for RARP compared to ORP [17, 18]. To account for learning curve effects, only surgeons with an adequate experience of a minimum of 50 previous cases have been included in the current analysis. In a recent learning curve analysis assessing RARP, Bravi et al. [19] assessed differences in PSM and BCR between surgeons with 10 and surgeons with 250 previous surgeries and found a 7.1% risk reduction for PSM. In a learning curve analysis for ORP by our working group, we found detectable learning curve effects regarding PSM and long-term continence for the first 750 and 300 procedures, respectively [9].

The largest study assessing outcomes after ORP and RARP included a total number of 10,790 patients from 1 very-high-volume institution. Regarding postoperative continence, higher early continence rates were found 1 week after catheter removal for the ORP subgroup (25.8 vs. 21.8%, *p* = 0.001), however, no significant differences could be found after 3 and 12 months after adjusting for age groups [4]. In the current study, we found higher continence rates based on the validated five-item ICIQ-SF questionnaire, which is hypothesized to be able to detect small differences in continence status more sensitively compared to pad usage.

In the current study, we focused on an “ideal” patient cohort, consisting of young, predominantly potent patients with favourable risk factors. We hypothesized that in these patients, postoperative HRQOL is of outstanding importance since they get to choose from several treatment options including active surveillance. In addition, adjuvant therapies are rarely needed in these patients and implications of adjuvant therapies on HRQOL outcomes can, therefore, be minimized [20]. We hypothesized that inclusion of patients with adequate preoperative erectile function and continence regardless of the pathological tumour stage have the potential to influence the postoperative HRQOL as well as functional outcomes and would, therefore, hamper the respective outcome analysis.

In the largest population-based study addressing HRQOL in prostate cancer patients to date, Downing et al. analysed PROMs in 11,000 men with localized or advanced prostate cancer. The authors found that, while most patients experience HRQOL comparable to the general population, in particular young patients < 55 years have worse HRQOL outcomes, especially if sexual function is deprived [21].

The strength of the current study is the well balanced and adequately large cohort, and the inclusion of preoperative baseline values. Based on propensity score matching, two cohorts of patients that did not differ in oncological as well as functional baseline characteristics were created.

For other uro-oncological entities, it has already been shown that preoperative HRQOL is an essential contributor in guidance of therapy decision-making [22] and it can be anticipated that this is also true for pre-RP patient counselling. In addition to previous studies, our analysis provides novel insights into the pre- and postoperative natural course of HRQOL of patients undergoing RP. Notably, we observe superior rates of good general HRQOL 3 months after ORP compared to RARP in univariate as well as multivariable analysis using well-accepted previously published cut-off values as surrogate endpoints [7]. We also provide data regarding the respective functioning and symptoms scores of the QLQ-C30 questionnaire. As mentioned above, we focused on patients with favourable clinical and tumour characteristics since these patients represent the subgroup with a particular need for equally favourable HRQOL outcomes.

Our study is the first one to report differences in general HRQOL between patients with favourable clinical and tumour characteristics after ORP and RARP. Importantly, these differences cannot be solely explained by differences in functional outcomes. First, our multivariable analysis could confirm differences in global health status scores even after adjustment for erectile function and urinary continence. Second, we found superior continence outcomes for ORP during the short-term follow-up on the one hand, but slightly inferior results regarding sexual outcomes on the other hand. Thus, in line with previous studies, no conclusive statement about superiority in functional outcomes can be drawn. One potential hypothesis for differences in short-term HRQOL between ORP and RARP patients might include aggressive marketing of RARP that might lead to unrealistic expectations of the patients that cannot be fulfilled and, therefore, potentially lead to a certain disappointment, especially in the early postoperative period. Our working group has already shown the perioperative patient counselling regarding realistic functional outcomes is essential for postoperative patient satisfaction [8]. Based on the results of the current study, this might be also true when it comes to realistic counselling of patients undergoing RARP.

Limitations of the current study are similar to previous comparative trials assessing outcomes after ORP and RARP. Even though only adequately experienced surgeons were included in the current study, learning curve effects cannot be excluded [2, 16]. In our patient cohort, median pre-analysis caseload was higher for ORP compared to RARP surgeons. However, we have shown in learning curve analyses from our department that a plateau regarding functional outcomes was reached after approximately 750 cases [9]. To further limit the effect of surgeons’ experience, a sensitivity analysis of the two most experienced surgeons in our patient cohort has been performed. Regarding further limitations, generalizability of our data is hampered by decrease in questionnaire response rates during the postoperative follow-up period. In the current study, we use the EORTC QLQ-C30 questionnaire to address patients HRQOL. Despite being not prostate cancer specific, this questionnaire provides robust results that can be compared with other entities as well as surgical procedures. To compensate the lack of domains that specifically address urinary and sexual symptoms, the validated IIEF-5 and ICIQ-SF questionnaires have been implemented in our analysis.

## Conclusions

In summary, we provide data from a well-balanced contemporary propensity score-matched patient cohort. Hereby, we focused on patients with favourable clinical and tumour characteristics since these patients represent the subgroup with the most urgent need for equally favourable HRQOL outcomes. We observed improved general HRQOL during the short-term follow-up for patients undergoing ORP compared to RARP in univariate as well as multivariable analysis. In the longer term follow-up, no significant differences in HRQOL as well as functional outcomes were observed.

## Electronic supplementary material

Below is the link to the electronic supplementary material.

Supplementary file1 (DOCX 13 kb)
