# Correction to: Health-related quality of life after open and robot-assisted radical prostatectomy in low- and intermediate-risk prostate cancer patients: a propensity score-matched analysis

**DOI:** 10.1007/s00345-021-03750-1

**Published:** 2021-07-01

**Authors:** Alexander Kretschmer, Robert Bischoff, Michael Chaloupka, Friedrich Jokisch, Thilo Westhofen, Philipp Weinhold, Frank Strittmatter, Armin Becker, Alexander Buchner, Christian G. Stief

**Affiliations:** Department of Urology, Ludwig-Maximilians-University, Marchioninistrasse 15, 81377 Munich, Germany

## Correction to: World Journal of Urology (2020) 38:3075–3083 10.1007/s00345-020-03144-9

The article “Health-related quality of life after open and robot-assisted radical prostatectomy in low- and intermediate-risk prostate cancer patients: a propensity score-matched analysis” written by Kretschmer, A., Bischoff, R., Chaloupka, M., Jokisch, F., Westhofen, T., Weinhold, P., Strittmatter, F., Becker, A., Buchner, A. and Stief, C.G. was originally published Online First without Open Access. After publication in volume 38, issue 12, pages 3075–3083 the author decided to opt for Open Choice and to make the article an Open Access publication. Therefore, the copyright of the article has been changed to © The Author(s) 2020 and the article is forthwith distributed under the terms of the Creative Commons Attribution 4.0 International License, which permits use, sharing, adaptation, distribution and reproduction in any medium or format, as long as you give appropriate credit to the original author(s) and the source, provide a link to the Creative Commons licence, and indicate if changes were made. The images or other third party material in this article are included in the article’s Creative Commons licence, unless indicated otherwise in a credit line to the material. If material is not included in the article’s Creative Commons licence and your intended use is not permitted by statutory regulation or exceeds the permitted use, you will need to obtain permission directly from the copyright holder. To view a copy of this licence, visit http://creativecommons.org/licenses/by/4.0.

The original article has been corrected.

