# Supplementary material for: Health-related quality of life after open and robot-assisted radical prostatectomy in low- and intermediate-risk prostate cancer patients: a propensity score-matched analysis
Source: World J Urol. 2020 Mar 4;38(12):3075–83. doi: 10.1007/s00345-020-03144-9 (PMC8249262; doi:10.1007/s00345-020-03144-9)
Supplement: Supplementary file 1 — Supplementary file1 (DOCX 13 kb) [file 345_2020_3144_MOESM1_ESM.docx]

**Supplementary material**

|  | **ORP** | **RARP** | **p** |
| --- | --- | --- | --- |
|  |  |  |  |
| **Good erectile function [IIEF5 ≥18] [%]*** |  |  |  |
| 3mo postop. | 29.0 | 33.3 | 0.669 |
| 12mo postop. | 38.6 | 55.9 | 0.100 |
| ≥24mo postop | 64.3 | 73.8 | 0.190 |
|  |  |  |  |
| **ICIQ-SF score [mean (SD)]** |  |  |  |
| Preoperative | 0.8 (2.6) | 0.7 (2.1) | 0.978 |
| 3mo postop. | 3.9 (3.4) | 5.3 (3.5) | **0.003** |
| 12mo postop. | 1.8 (3.1) | 2.6 (3.1) | 0.072 |
| ≥24mo postop | 1.9 (3.1) | 1.9 (3.0) | 0.949 |
|  |  |  |  |
| **Good HRQOL [QLQ-C30 GLH >70] [%]** |  |  |  |
| Preoperative | 65.2 | 57.3 | 0.131 |
| 3mo postop. | 55.7 | 29.5 | **<0.001** |
| 12mo postop. | 63.2 | 48.6 | 0.091 |
| ≥24mo postop | 56.8 | 61.0 | 0.819 |

**Supplementary table 1** Functional outcomes after open retropubic (ORP) and robot-assisted radical prostatectomy (RARP) (ICIQ-SF = International Consultation of Incontinence Short Form; IIEF-5 = International Index of Erectile Function; GLH = global health status; HRQOL = health-related quality of life; SD = standard deviation).
